# Undergraduate dental students’ perspective of online learning and their physical and mental health during COVID-19 pandemic

**DOI:** 10.1371/journal.pone.0270091

**Published:** 2022-06-16

**Authors:** Enas Abdalla Etajuri, Noorhayati Raja Mohd, Zahra Naimie, Norasmatul Akma Ahmad

**Affiliations:** 1 Department of Restorative Dentistry, Faculty of Dentistry, University of Malaya, Kuala Lumpur, Malaysia; 2 Deans Office, Faculty of Dentistry, University of Malaya, Kuala Lumpur, Malaysia; Endeavour College of Natural Health, AUSTRALIA

## Abstract

**Background and objective:**

COVID-19 pandemic continuously spread exacerbating global concerns. It had impacted all life aspects such as social, cultural, economic and education. This study assess the impact of COVID-19 pandemic on undergraduate dental students at Faculty of Dentistry, University of Malaya. The objective of this study is to evaluate the impact of the pandemic on physical and mental health of undergraduate dental students.

**Methods:**

This is a cross-sectional study. An online questionnaire was administrated to 150 undergraduate dental students. The questionnaire included questions related to students’ demographic data, their concerns on academic achievement, their opinion on institution response, and the impact of the crisis on their mental and physical health. SPSS software v26.0 was used to analyse the collected data.

**Results:**

A total of 147 respondents participated in the study. About 66% of the students felt comfortable in adapting to the new technology while 85.7% were concerned about the quality of online learning. Almost all students 98.6% expressed their doubts on the ability to pass the competency exams and to graduate on time, with only 49.7% agreed that clinical experience was effectively delivered through online classes. Most students were also concerned on the impact of the pandemic on their physical and emotional health 85.8% and 76.9% respectively.

**Conclusions:**

The findings of our study highlight the positive adaptation of the students to online learning and using technology. On the other hand, the study indicates that the COVID-19 pandemic brings negative impacts on our students’ physical and mental health.

## Background

The continuous evolution of the coronavirus disease-2019 (COVID-19) pandemic arguably led to the worst global health disaster in the current century, owing to the quick spread and increased morbidity and mortality rates [1]. Daily activities were massively interrupted all around the world despite the efforts that were put forward to mitigate the disease outbreaks.

The pandemic had a huge impact on mental and physical health worldwide, the appearance of the symptoms such as, post-traumatic stress disorder (PTSD), depression, stress, and anxiety were reported among the population globally. The high prevalence of psychological symptoms was linked to some risk factors include younger age, students, joblessness, and regular exposure to news and rumours regarding COVID-19 [2]. Social and physical distancing along with self-isolation and lockdowns also have significantly limited face-to-face social interaction with others [3–5], considering the nature of dentistry course, dental students are prone to experience similar emotional stress and depression.

In general, educational institutions and dental institutions in particular were no exception to this interruption as various schools were closed and forced to rapidly switched from face-to-face teaching to online/virtual learning. In dentistry, although theoretical aspects can be adequately covered virtually via technology advancement and online educational content [6] the practical aspect (clinical teaching) will still suffer. In dental education, both clinical and didactic teaching are equally important. However, no online session can replace the students’ experience with patients in the clinics [7]. Practical training on manikins and patient treatment are indispensable in dental education, [8].

To safeguard the standard of graduating dentist, all dental institutions in the world are subjected to comply to a standard endorsed by their own regulatory bodies. In Malaysia, a governing body called Malaysian Qualifying Agency (MQA) coordinates the dental education system, and all dental schools are required to adhere to the criteria set by them. The dental undergraduate program consists of five years in which the first two years are focused on the basic medical science and preclinical dental education and training. From year 3 to 5 the students are required to join clinical sessions and fulfil specific clinical requirements to be able to set for competency‐based assessment in all dental disciplines before graduation.

Dental graduates must be clinically competent at the end of their training and this is assessed by sets of competency tests and exams. Competencies describe the students’ understanding, skills and professional values that are needed for the unsupervised dental clinical practice as a qualified dentist [9]. Due to COVID-19 pandemic, Malaysian dental institutions have been revising their approach to teaching and learning in order to minimize the impact of COVID-19 pandemic on dental education. The minimal clinical experience (MCE) standard to graduate were also reviewed by the Deans caucus and endorsed by the Malaysian Dental Council accordingly due to this pandemic.

The integration of technology into dental education has been steadily progressive in Malaysia. E-learning has been one of the teaching modes even before the pandemic and has contributed significantly to the teaching and learning activities in the dental institutions. However due to this COVID-19 pandemic, with risk of spreading the virus through contacts in the polyclinics, face-to-face approach were shifted to E-learning. Online learning/distance learning drastically obtaining more popularity and act as the sole option for the educators to ensure that teaching and learning continues and is not being impaired. Obviously, the shift towards online learning was accelerated by this unprecedented pandemic, while both academic staff and students might not be fully prepared on such teaching and learning modality. This sudden shift from face-to-face to online learning creates a lot of challenges in an era of teaching and learning for institutions that are involved with health education, namely limited access to Internet, poor interactions with the teachers [10] and many more. However, the impact of this shift in teaching modality on dental students is yet to be fully explored including their physical and emotional health.

The undergraduate dental student’s perspectives towards the impact of the COVID-19 pandemic on their education as well as their mental and physical health is essential in preparing a robust dental curriculum that can withstand any future challenges. Thus, this study aimed to evaluate the impact of the pandemic on the education of undergraduate dental students including the effect on their physical and mental health. While the pandemic still evolving, the outcome of this study may be beneficial to improve the practice guidelines, and to help the students in adapting to the new norm by providing supportive and motivation guidance whenever possible. Furthermore, the findings can assist policy makers in view of any future disasters.

## Materials and methods

### Sample

This was a cross-sectional study conducted in Faculty of Dentistry, University of Malaya. All clinical year students (year III, year IV and year V) were invited to take the survey as they have been mainly affected by cancellations of clinical sessions due to COVID-19 pandemic. A Google Form link to the survey was sent to 152 students using their email addresses followed by a reminding email on the subsequent week. The survey was conducted on June 2021 during the Malaysian nationwide total lockdown period that extended until 15^th^ October 2021. Ethical approval DF RD2108/0031 was obtained from the Ethics committee at Faculty of Dentistry, University of Malaya.

The objectives of the study, researchers contacts information, the stipulated time needed as well as the deadline to complete the survey were stated in the sent email. The time needed to complete the survey is between 8–10 minutes and data was collected within two weeks. An agreement statement that the participation is voluntary was provided on the first page of the Google form. The participation was anonymously, and the consent was obtained by clicking on the agree button. A total of 147 students completed and submitted the survey.

### Instruments

A questionnaire (S1 Appendix) adapted from Hung et al. [11] was used for this research. Items related to assessment of students’ concerns about their academic achievement, institution response to the pandemic, and their physical and mental health’s were used to evaluate the research objectives.

The authors of this paper had modified and validated the survey. A modification was done on the format of the questions The modification was also done on the questions related to the students’ academic concerns where one question asking about the student ability to pass dental board exam was slightly modified to ask about the ability to pass the clinical competency exam. This is because the competency tests are the main assessment exams for the students of dental faculty, University of Malaya to graduate. Another question was asking if the students are willing to take shorter winter break to make up for the educational experience lost, it was modified to ask about shorter semester break according to the terminology used in Malaysia. Two questions were eliminated from the survey due to inapplicability in the Malaysian education system. The questions were asking students if they are welling to cancel their travels and to attend 12 hours per day to overcome the missing session. Prior to the main data collection, a pilot study was conducted to 10 dental students from other local university to ensure the survey items are understandable and clear. The pilot test results were compiled and there was no further modification to the questionnaire based on the participants responses.

The questionnaire consists of three sections with close-ended questions using 5-points Likert scale aiming to collect relevant data from the participants. Table 1 indicated the sections of the modified questionnaire used in this study.

**Table 1:** Survey sections.

Sec	Area assessed	Number of Items	Scale
1	Demography such as age, gender, academic year, place of current residence	4	Close ended
2	Students’ perspective on: • Their academic achievement • Their institution measures to halt the sessions and converting to online learning due to COVID-19	12	Likert scales
3	Students’ perspective on: Students physical and mental health due to COVID-19	17	Likert scales
Total		**33**	

For this study, the collected data was analysed using the Statistical Package for the Social Sciences SPSS® (v26.0, SPSS Inc., IBM Corp, Armonk, NY, USA), and descriptive analysis was performed (S2 and S3 Appendices).

## Results

### Demographic data

A total of 147 responses to the survey were collected. Among it, 24.5% were male, and 75.5% were female. There were 64.6% students who resided in the university hostel and 35.4% resided at home. Among the 147 students, 32.7% were third year students, 34% were fourth year students, and 33.3% were fifth year students.

### Assessment of academic achievement concerns

Most students (66%) felt comfortable adapting to the new technology, while 27.2% reported feeling neutral to the technology shift. Only 0.7% of the students felt uncomfortable in adapting to the technology shift. However, most students (85.7%) were concerned about the quality of online teaching. In the matter of academic achievement, 40.2% of the students were often or always felt difficult focusing on their faculty work, and more than half of students (53%) faced difficulty getting the motivation to study. The vast majority (94.5%) of the students were concerned about the likelihood of graduating on time, and 98.6% of them were worried about passing their clinical competency exams on time. Despite the very high concern on the ability to graduate on time, only (63.3%) were willing to make up their clinical and practical experience lost by taking shorter semester breaks. About 32.6% of the students were ready to attend the faculty classes six days a week after school reopen.

### Assessment of institution response

Regarding the faculty responses to COVID-19, 42.2% of the students found it as effective, and 40.1% viewed it neutrally. However, almost half of the students (50%) thought that the faculty transition from face-to-face to online courses was effective, and only 11.6% rated it as ineffective. Regarding the lecturer’s effectiveness in providing an online course, more than two-thirds (70.1%) of the students rated it as effective. However, almost half of the students (49.7%) rated their lecturer as effective in providing clinical experience during online classes.

### Assessment of physical health

The majority of the students (85.8%) were concerned about the effect of COVID-19 on their physical health. Over one-third of students (38.1%) suffered restless sleep, with 26.5% felt anxious about being infected with the virus. Almost three quarters of the students (74.1%) were also concerned about contracting the virus while providing treatment in the clinic. 60.5% of the students expressed their concern about contracting the virus from attending face-to-face classes in the class room while 67.3% of the students were concern about contracting it from interacting with people at the faculty building.

### Assessment of mental health

The vast majority of the students (76.9%) were concerned about their emotional health, with 47% found it difficult to control important things in their life. Many students (43.5%) often or always felt they could not cope with all of the things they had to do, and 36.7% felt angry when their things turned out of control. More than half of the students (57.8%) felt stressed and anxious (57.9%) because of the uncertainly about how long the current crisis will last. 26.5% of students often or always have felt depressed, and 29.3% sometimes experienced depression. Over two-thirds of the students were concerned about their accommodation issues after the faculty reopens. Generally, all students (93.9%) were worried about the wellbeing of their relatives, with 74.9% concerned about their social connections. Some of the students (24.5%) even felt lonely during the pandemic outbreak.

## Discussion

COVID-19 SARS-CoV-2 is a new virus that has had a global harmful effect since it was first revealed in December 2019. It has been reported that human-to-human transmission of the virus probably occurs through the exposure to respiratory droplets and aerosols of infected individuals [12]. Furthermore, the SARS-CoV-2 transmission also includes the oral mucous tissues, gastrointestinal tract (fecal–oral) and the eyes [13, 14]. In has been reported in a recent study that the survival rate of SARS-CoV-2 in aerosols can be as long as three hours, and the virus stability on stainless steel and plastic is up to three days (72h) [12].

Routine dental procedures involving ultrasonic and headpieces instruments are generating bioaerosols that contains oral fluid and microbial deposits. These aerosols were reported to be associated in the transmission of influenza and SARS-CoV virus [15]. Instrument contamination from SARS-CoV virus causing self-inoculation throughout mucous tissues is also possible [16, 17] and, therefore, poses a higher risk of disease contracting to dental care providers. It has been reported by the Occupational Safety and Health Administration (OSHA) in their guide to prepare workplaces for COVID-19 that dentistry aligned under the very high-risk field [18]. With this description in mind, all dental care providers should take extra precautions to avoid the risk of SARS-CoV-2 transmission while delivering dental treatment to patients.

The magnitude of disease spread can be worrying especially in dental education institutions. The higher risk is related to the huge amount of aerosol generation that effects more individuals at the interconnected dental teaching units [19], the presence of large number of patients in a confined space, and the insufficient/scant adherence to standard operating procedures (SOP) by undergraduate students. Due to the aforementioned factors, many dental education institutions shifted to online learning and suspended all face-to-face classes [11, 20–23].

There are several published papers studied the efficiency of online learning during the pandemic [24, 25]. Online learning is not commonly used in Asia before the COVID-19 pandemic and the unexpected and sudden shift to online teaching took some time by dental institutions to prepare for it [26]. However, to date there is no study on the student’s perspective and adaptation to online learning during the lock down period in Malaysian dental institutions.

Adapting to online learning was vital especially during the postponement of face-to-face sessions within the lockdown period to achieve the educational goals [27]. The current study involved the clinical year students only, as their clinical skills depends more on the face-to-face teaching and on the real clinical practice with patient interaction, in addition to the effect of the halt of clinical practice that leads to delay in fulfilling their clinical requirements and passing the competency exams. In comparison to the pre-clinical years where the curriculum depends more on the theoretical part. Many of our students expressed their satisfaction with the adaptation to the online learning process. This depends on the students and academic staff skills on using information technology. Chang et al. studied the online learning effect on undergraduate dental students in 13 Asian dental institutions during the pandemic, they found that students were satisfied and able to adapt to online learning [26]. On the other hand, it has been reported that the students are faster in adapting to online learning comparing to their senior lecturer [28].

Most of our students were concerned about the quality of online courses at our institution. In addition, more than half of the respondents had difficulty to focus on their faculty work and feel demotivated. This result could be related to the nature of the dental field that mainly depends on face-to-face clinical and practical sessions. Our results are comparable with another study where a lower level of students’ satisfaction to online learning was reported, and corelated to the difficulty of students’ communication with the academic instructors and colleagues. Students time management, readiness and ability for being focused for long online sessions are also challenging factors [23].

Many students reported that the faculty responded to the COVID-19 pandemic positively. They rated an effective shift to online classes and satisfaction towards the performance of the lecturers in delivering the online courses. Only half of the students found that their lecturers were able to provide clinical knowledge through online sessions and this is expected as practical and clinical are indispensable in dental education [8]. However, 49.7% of the students only were satisfied with the clinical knowledge delivered through on-line classes. This fact is related to the limited manual training and the lack of interaction with patients. It highlights the necessity to develop new approaches, such as providing demonstration, videotaping cases by faculty academic staff. This finding was also reported by other study where many students preferred physical classes over online and simulated training classes [26].

In-deed the online learning cannot fully replace the face-to-face clinical practice, however the academician had conducted different live online activities, such as case-based learning, case-based reasoning, and group interactive discussions between the lecture and the entire class. Furthermore, many clinical-based education materials and demonstrations videotaping were provided to the student through the university E-learning platform (UM eSPECTRUM) and on the faculty YouTube channel to strengthen students’ clinical background. Recently, innovative technology such as virtual reality (VR) is widely used in dental education [29]. It has been reported that students could remember more knowledge and successfully apply what they had practiced in VR trainings [30]. This modern technology will enable the educational institutions to successfully provide distance education courses.

Dentistry is a stressful course by itself. The leading causes of stress among dental students involve heavy clinical and practical training, management of challenging clinical cases, the need to pass clinical and academic requirements, and lecturers and patients’ interactions [31].

In addition to the factors above, dental students now face additional pressures due to the high risk of contracting the disease among teachers and students once school reopens [26]. This was supported by our results where majority of our participants were concerned about their physical health. Accordingly, dental education institutions should adopt a prevention guideline for the safety of academician, students, and patients.

In July 2020, the Association for Dental Education, Asia Pacific (ADEAP) published a detailed safety guidelines for safe dental education during the pandemic [32]. Similarly, the Faculty of Dentistry, University of Malaya, has its COVID-19 Task Force and guidelines in ensuring the safety of patients, students, academicians and the supporting staff. The students, staff and patients were required to answer a screening survey before entering the faculty building. Individuals who have a covid-19 symptoms or have a close contact with a positive case of COVID-19 during the last 14 days were not permitted to enter the faculty building. All dental clinic cubicle were enclosed, a PPE donning and doffing locations were prepared, the students were allowed to provide treatment for only one patient per cubicle during the clinical session, and all dental materials and equipment’s were prepared at an enclosed dedicated cubicle to minimise the movement and interactions between students and staff. This protocol should provide additional assurance for the student on the limited risk of being contracted with the virus.

Several studies were conducted to evaluate the stress level perceived by dental students [31, 33, 34], it showed that the stress effects students’ performance and results in poorer levels of physical and mental health [34]. In addition, it leads to emotional exhaustion and depersonalization [35]. Stress can negatively affect dental students. To date, little has been published about stress related to COVID-19 crisis. The current study provides actual evidence on stress faced by the students during this pandemic. Many students reported concerns about their emotional health, being stressed and felt things were getting out of control. They also worried about their social connections and loneliness. This might be the consequences due to the movement control order as most students have lost their social networking specially when most of them were staying in the university hostel, and they were not able to meet their families for a subsequently a long period of time. Faculty support during this period was found to be very efficient as consistent communication between the faculty management and students were observed. An online meeting between all the students with their mentors were also carried out to discuss their needs and provide support required and eliminate their anxiety. The limitation of this study is that the survey was conducted during the lockdown period. As there was no previous data on the student stress related to the nature of their study field, we should highlight that other factor that leads to the increase of student stress could has an effect on the students responses.

## Conclusion

The COVID-19 pandemic was perceived to have impacted the undergraduate dental students in many ways including their academic achievements, social, mental and physical health. The study conclude that undergraduate dental students were able to adapt to online learning, but they were concerned about their physical and mental health. The current findings highlight the need of institutions to fine-tuned their shift into online learning by taking into account the input of the students, to adjust their curriculum accordingly taking into consideration the loss of clinical sessions, the safety of all patients, students and staff alike as well as the mental and physical health of the students being stranded away from their families and lacking social interactions due to the social distancing requirement.

## Supporting information

S1 Appendix Questionnaires. (DOCX)

S2 Appendix (DOCX)

S3 Appendix (DOCX)
