# Supplementary material for: Undergraduate dental students’ perspective of online learning and their physical and mental health during COVID-19 pandemic
Source: PLoS One. 2022 Jun 16;17(6):e0270091. doi: 10.1371/journal.pone.0270091 (PMC9491624; doi:10.1371/journal.pone.0270091)
Supplement: S1 Appendix — (DOCX) [file pone.0270091.s001.docx]

**Questionnaires**

**Section 1: Demographic Data**

| Question | Options | | |
| --- | --- | --- | --- |
| Age | Participant to type | | |
| Gender | Male | Female |  |
| Study year | BDS year 3 | BDS year 4 | BDS year 5 |
| Place of resident | Home | Hostel | |

**Section 2: Assessment of academic concerns and institutional responses**

| Questions | | Options | | | | |
| --- | --- | --- | --- | --- | --- | --- |
| **Academic Concern** | | | | | | |
| 1 | How comfortable are you in adapting to new technology? | Ineffective | Slightly ineffective | Neutral | Slightly effective | Effective |
| 2 | In light to COVID-19, are you concerned about the quality of your online courses? | Not Concerned | A little concerned | Concerned | Very concerned | Extremely concerned |
| 3 | In light to COVID-19, how often did you feel difficult to focus on faculty work? | Never | Occasionally | Sometimes | Often | Always |
| 4 | In light to COVID-19, how often did you feel difficult to find motivation to study? | Never | Occasionally | Sometimes | Often | Always |
| 5 | In light to COVID-19, are you concerned about the like hood that you will complete your degree program on time? | Not Concerned | A little concerned | Concerned | Very concerned | Extremely concerned |
| 6 | In light to COVID-19, are you concerned about passing your clinical competency exam on a timely manner? | Not Concerned | A little concerned | Concerned | Very concerned | Extremely concerned |
| 7 | In light to COVID-19, would you be welling to make up for the educational experience lost so that you can graduate on time by taking a shorter semester break after school reopen? | Not at all | Slightly | Probably | Very likely | Definitely |
| 8 | In light to COVID-19, would you be welling to make up for the educational experience lost so that you can graduate on time by attending school 6 days per week after school reopen? | Not at all | Slightly | Probably | Very likely | Definitely |
| **Institutional responses** | | | | | | |
| 9 | In light to COVID-19, how effective do you think your faculty overall response to COVID-19 ? | Ineffective | Slightly ineffective | Neutral | Slightly effective | Effective |
| 10 | In light to COVID-19, how effective do you think your faculty was in transitioning to on-line courses? | Ineffective | Slightly ineffective | Neutral | Slightly effective | Effective |
| 11 | In light to COVID-19, how effective do you think your lecturer were in teaching online courses during faculty closure? | Ineffective | Slightly ineffective | Neutral | Slightly effective | Effective |
| 12 | In light to COVID-19, how effective do you think your lecturer were in providing clinical experience during faculty closure? | Ineffective | Slightly ineffective | Neutral | Slightly effective | Effective |

**Section 3: Physical and mental health concerns**

| Questions | | Options | | | | |
| --- | --- | --- | --- | --- | --- | --- |
| **Physical health concern** | |  | | | | |
| 1 | In light to COVID-19, are you concerned about your physical health? | Not Concerned | A little concerned | Concerned | Very concerned | Extremely concerned |
| 2 | In light to COVID-19, how often did you feel your sleep was restless? | Never | Occasionally | Sometimes | Often | Always |
| 3 | In light to COVID-19, how often did you feel anxious that you might get infected with COVID-19 virus? | Never | Occasionally | Sometimes | Often | Always |
| 4 | In light to COVID-19, are you concerned about contracting COVID -19 from providing patient care in the clinics? | Not Concerned | A little concerned | Concerned | Very concerned | Extremely concerned |
| 5 | In light to COVID-19, are you concerned about contracting COVID -19 from attending classes in the faculty? | Not Concerned | A little concerned | Concerned | Very concerned | Extremely concerned |
| 6 | In light to COVID-19, are you concerned about contracting COVID -19 from interacting with people in the faculty building? | Not Concerned | A little concerned | Concerned | Very concerned | Extremely concerned |
| **Mental health concern** | | | | | | |
| 1 | In light to COVID-19, are you concerned about about-your emotional health? | Not Concerned | A little concerned | Concerned | Very concerned | Extremely concerned |
| 2 | In light to COVID-19, how often did you feel you were unable to control important things in your life? | Never | Occasionally | Sometimes | Often | Always |
| 3 | In light to COVID-19, how often did you feel you could not cope with all of the things that you had to do? | Never | Occasionally | Sometimes | Often | Always |
| 4 | In light to COVID-19, how often did you feel how often do you feel angry because things were outside of your control? | Never | Occasionally | Sometimes | Often | Always |
| 5 | In light to COVID-19, how often did you feel stressed? | Never | Occasionally | Sometimes | Often | Always |
| 6 | In light to COVID-19, how often did you feel anxious regarding the uncertainly about how long the current crisis will last? | Never | Occasionally | Sometimes | Often | Always |
| 7 | In light to COVID-19, how often did you feel depressed? | Never | Occasionally | Sometimes | Often | Always |
| 8 | In light to COVID-19, are you concerned about your housing situation after faculty re-opens? | Not Concerned | A little concerned | Concerned | Very concerned | Extremely concerned |
| 9 | In light to COVID-19, are you concerned about the well being of your family? | Not Concerned | A little concerned | Concerned | Very concerned | Extremely concerned |
| 10 | In light to COVID-19, are you concerned about your social connection? | Not Concerned | A little concerned | Concerned | Very concerned | Extremely concerned |
| 11 | In light to COVID-19, how often did you feel lonely? | Never | Occasionally | Sometimes | Often | Always |
