# Supplementary material for: Undergraduate dental students’ perspective of online learning and their physical and mental health during COVID-19 pandemic
Source: PLoS One. 2022 Jun 16;17(6):e0270091. doi: 10.1371/journal.pone.0270091 (PMC9491624; doi:10.1371/journal.pone.0270091)
Supplement: S2 Appendix — (DOCX) [file pone.0270091.s002.docx]

**Appendix I**

**Section 1: Demographic data:**

**Section 2a : Assessment of academic concerns (8 questions)**

**Section 2b : Assessment institutional responses (4 questions)**

**Section 3a: Physical health concerns (6 questions):**

**Section 3b: Mental health concerns (11 questions):**
