# Supplementary material for: Undergraduate dental students’ perspective of online learning and their physical and mental health during COVID-19 pandemic
Source: PLoS One. 2022 Jun 16;17(6):e0270091. doi: 10.1371/journal.pone.0270091 (PMC9491624; doi:10.1371/journal.pone.0270091)

Appendix III

**Frequencies**

| **Age** | | | | | |
| --- | --- | --- | --- | --- | --- |
|  | | Frequency | Percent | Valid Percent | Cumulative Percent |
| Valid | 21y | 4 | 2.3 | 2.7 | 2.7 |
|  | 22y | 53 | 31.0 | 36.1 | 38.8 |
|  | 23y | 46 | 26.9 | 31.3 | 70.1 |
|  | 24y | 42 | 24.6 | 28.6 | 98.6 |
|  | 25y | 2 | 1.2 | 1.4 | 100.0 |
|  | Total | 147 | 86.0 | 100.0 |  |
| Missing | System | 24 | 14.0 |  |  |
| Total | | 171 | 100.0 |  |  |

| **Gender** | | | | | |
| --- | --- | --- | --- | --- | --- |
|  | | Frequency | Percent | Valid Percent | Cumulative Percent |
| Valid | female | 111 | 64.9 | 75.5 | 75.5 |
|  | male | 36 | 21.1 | 24.5 | 100.0 |
|  | Total | 147 | 86.0 | 100.0 |  |
| Missing | System | 24 | 14.0 |  |  |
| Total | | 171 | 100.0 |  |  |

| **Study year** | | | | | |
| --- | --- | --- | --- | --- | --- |
|  | | Frequency | Percent | Valid Percent | Cumulative Percent |
| Valid | Year3 | 48 | 28.1 | 32.7 | 32.7 |
|  | Year4 | 50 | 29.2 | 34.0 | 66.7 |
|  | Year5 | 49 | 28.7 | 33.3 | 100.0 |
|  | Total | 147 | 86.0 | 100.0 |  |
| Missing | System | 24 | 14.0 |  |  |
| Total | | 171 | 100.0 |  |  |

| **Place of residence** | | | | | |
| --- | --- | --- | --- | --- | --- |
|  | | Frequency | Percent | Valid Percent | Cumulative Percent |
| Valid | Home | 52 | 30.4 | 35.4 | 35.4 |
|  | Hostel | 95 | 55.6 | 64.6 | 100.0 |
|  | Total | 147 | 86.0 | 100.0 |  |
| Missing | System | 24 | 14.0 |  |  |
| Total | | 171 | 100.0 |  |  |

| **1. How comfortable are you in adapting to new technology?** | | | | | |
| --- | --- | --- | --- | --- | --- |
|  | | Frequency | Percent | Valid Percent | Cumulative Percent |
| Valid | Ineffective | 1 | .6 | .7 | .7 |
|  | Slightly ineffective | 9 | 5.3 | 6.1 | 6.8 |
|  | Neutral | 40 | 23.4 | 27.2 | 34.0 |
|  | Slightly effective | 66 | 38.6 | 44.9 | 78.9 |
|  | Effective | 31 | 18.1 | 21.1 | 100.0 |
|  | Total | 147 | 86.0 | 100.0 |  |
| Missing | System | 24 | 14.0 |  |  |
| Total | | 171 | 100.0 |  |  |

| **2.In the light to COVID-19, are you concerned about the quality of your online courses?** | | | | | |
| --- | --- | --- | --- | --- | --- |
|  | | Frequency | Percent | Valid Percent | Cumulative Percent |
| Valid | Not Concerned | 3 | 1.8 | 2.0 | 2.0 |
|  | A little concerned | 18 | 10.5 | 12.2 | 14.3 |
|  | Concerned | 50 | 29.2 | 34.0 | 48.3 |
|  | Very concerned | 48 | 28.1 | 32.7 | 81.0 |
|  | Extremely concerned | 28 | 16.4 | 19.0 | 100.0 |
|  | Total | 147 | 86.0 | 100.0 |  |
| Missing | System | 24 | 14.0 |  |  |
| Total | | 171 | 100.0 |  |  |

| **3.In the light to COVID-19, how often did you feel difficult to focus on faculty work?** | | | | | |
| --- | --- | --- | --- | --- | --- |
|  | | Frequency | Percent | Valid Percent | Cumulative Percent |
| Valid | Never | 2 | 1.2 | 1.4 | 1.4 |
|  | Occasionally | 23 | 13.5 | 15.6 | 17.0 |
|  | Sometimes | 63 | 36.8 | 42.9 | 59.9 |
|  | Often | 42 | 24.6 | 28.6 | 88.4 |
|  | Always | 17 | 9.9 | 11.6 | 100.0 |
|  | Total | 147 | 86.0 | 100.0 |  |
| Missing | System | 24 | 14.0 |  |  |
| Total | | 171 | 100.0 |  |  |

| **4.n the light to COVID-19, how often did you feel difficult to find motivation to study?** | | | | | |
| --- | --- | --- | --- | --- | --- |
|  | | Frequency | Percent | Valid Percent | Cumulative Percent |
| Valid | Never | 2 | 1.2 | 1.4 | 1.4 |
|  | Occasionally | 26 | 15.2 | 17.7 | 19.0 |
|  | Sometimes | 41 | 24.0 | 27.9 | 46.9 |
|  | Often | 44 | 25.7 | 29.9 | 76.9 |
|  | Always | 34 | 19.9 | 23.1 | 100.0 |
|  | Total | 147 | 86.0 | 100.0 |  |
| Missing | System | 24 | 14.0 |  |  |
| Total | | 171 | 100.0 |  |  |

| **5.In the light to COVID-19, are you concerned about the like hood that you will complete your degree program on time?** | | | | | |
| --- | --- | --- | --- | --- | --- |
|  | | Frequency | Percent | Valid Percent | Cumulative Percent |
| Valid | Not Concerned | 1 | .6 | .7 | .7 |
|  | A little concerned | 7 | 4.1 | 4.8 | 5.4 |
|  | Concerned | 23 | 13.5 | 15.6 | 21.1 |
|  | Very concerned | 49 | 28.7 | 33.3 | 54.4 |
|  | Extremely concerned | 67 | 39.2 | 45.6 | 100.0 |
|  | Total | 147 | 86.0 | 100.0 |  |
| Missing | System | 24 | 14.0 |  |  |
| Total | | 171 | 100.0 |  |  |

| **6.In the light to COVID-19, are you concerned about passing your clinical competency exam on a timely manner?** | | | | | |
| --- | --- | --- | --- | --- | --- |
|  | | Frequency | Percent | Valid Percent | Cumulative Percent |
| Valid | A little concerned | 2 | 1.2 | 1.4 | 1.4 |
|  | Concerned | 11 | 6.4 | 7.5 | 8.8 |
|  | Very concerned | 45 | 26.3 | 30.6 | 39.5 |
|  | Extremely concerned | 89 | 52.0 | 60.5 | 100.0 |
|  | Total | 147 | 86.0 | 100.0 |  |
| Missing | System | 24 | 14.0 |  |  |
| Total | | 171 | 100.0 |  |  |

| **7.In the light to COVID-19, would you be welling to make up for the educational experience lost so that you can graduate on time by taking a shorter semester break after school reopen?** | | | | | |
| --- | --- | --- | --- | --- | --- |
|  | | Frequency | Percent | Valid Percent | Cumulative Percent |
| Valid | Not at all | 4 | 2.3 | 2.7 | 2.7 |
|  | Slightly | 7 | 4.1 | 4.8 | 7.5 |
|  | Probably | 43 | 25.1 | 29.3 | 36.7 |
|  | Very likely | 46 | 26.9 | 31.3 | 68.0 |
|  | Definitely | 47 | 27.5 | 32.0 | 100.0 |
|  | Total | 147 | 86.0 | 100.0 |  |
| Missing | System | 24 | 14.0 |  |  |
| Total | | 171 | 100.0 |  |  |

| **8. In the light to COVID-19, would you be welling to make up for the educational experience lost so that you can graduate on time by attending school 6 days per week after school reopen?** | | | | | |
| --- | --- | --- | --- | --- | --- |
|  | | Frequency | Percent | Valid Percent | Cumulative Percent |
| Valid | Not at all | 32 | 18.7 | 21.8 | 21.8 |
|  | Slightly | 24 | 14.0 | 16.3 | 38.1 |
|  | Probably | 43 | 25.1 | 29.3 | 67.3 |
|  | Very likely | 30 | 17.5 | 20.4 | 87.8 |
|  | Definitely | 18 | 10.5 | 12.2 | 100.0 |
|  | Total | 147 | 86.0 | 100.0 |  |
| Missing | System | 24 | 14.0 |  |  |
| Total | | 171 | 100.0 |  |  |

| **9. In the light to COVID-19, how effective do you think your faculty overall response to COVID-19 ?** | | | | | |
| --- | --- | --- | --- | --- | --- |
|  | | Frequency | Percent | Valid Percent | Cumulative Percent |
| Valid | Ineffective | 5 | 2.9 | 3.4 | 3.4 |
|  | Slightly ineffective | 21 | 12.3 | 14.3 | 17.7 |
|  | Neutral | 59 | 34.5 | 40.1 | 57.8 |
|  | Slightly effective | 43 | 25.1 | 29.3 | 87.1 |
|  | Effective | 19 | 11.1 | 12.9 | 100.0 |
|  | Total | 147 | 86.0 | 100.0 |  |
| Missing | System | 24 | 14.0 |  |  |
| Total | | 171 | 100.0 |  |  |

| **10. In the light to COVID-19, how effective do you think your faculty was in transitioning to on-line courses?** | | | | | |
| --- | --- | --- | --- | --- | --- |
|  | | Frequency | Percent | Valid Percent | Cumulative Percent |
| Valid | Ineffective | 4 | 2.3 | 2.7 | 2.7 |
|  | Slightly ineffective | 13 | 7.6 | 8.8 | 11.6 |
|  | Neutral | 56 | 32.7 | 38.1 | 49.7 |
|  | Slightly effective | 49 | 28.7 | 33.3 | 83.0 |
|  | Effective | 25 | 14.6 | 17.0 | 100.0 |
|  | Total | 147 | 86.0 | 100.0 |  |
| Missing | System | 24 | 14.0 |  |  |
| Total | | 171 | 100.0 |  |  |

| **11. In the light to COVID-19, how effective do you think your lecturer were in teaching online courses during faculty closure?** | | | | | |
| --- | --- | --- | --- | --- | --- |
|  | | Frequency | Percent | Valid Percent | Cumulative Percent |
| Valid | Ineffective | 1 | .6 | .7 | .7 |
|  | Slightly ineffective | 1 | .6 | .7 | 1.4 |
|  | Neutral | 42 | 24.6 | 28.6 | 29.9 |
|  | Slightly effective | 68 | 39.8 | 46.3 | 76.2 |
|  | Effective | 35 | 20.5 | 23.8 | 100.0 |
|  | Total | 147 | 86.0 | 100.0 |  |
| Missing | System | 24 | 14.0 |  |  |
| Total | | 171 | 100.0 |  |  |

| **12.In the light to COVID-19, how effective do you think your lecturer were in providing clinical experience during faculty closure? [Score]** | | | | | |
| --- | --- | --- | --- | --- | --- |
|  | | Frequency | Percent | Valid Percent | Cumulative Percent |
| Valid | Ineffective | 2 | 1.2 | 1.4 | 1.4 |
|  | Slightly ineffective | 15 | 8.8 | 10.2 | 11.6 |
|  | Neutral | 57 | 33.3 | 38.8 | 50.3 |
|  | Slightly effective | 52 | 30.4 | 35.4 | 85.7 |
|  | Effective | 21 | 12.3 | 14.3 | 100.0 |
|  | Total | 147 | 86.0 | 100.0 |  |
| Missing | System | 24 | 14.0 |  |  |
| Total | | 171 | 100.0 |  |  |

| **1.In the light to COVID-19, are you concerned about your physical health?** | | | | | |
| --- | --- | --- | --- | --- | --- |
|  | | Frequency | Percent | Valid Percent | Cumulative Percent |
| Valid | Not Concerned | 5 | 2.9 | 3.4 | 3.4 |
|  | A little concerned | 16 | 9.4 | 10.9 | 14.3 |
|  | Concerned | 43 | 25.1 | 29.3 | 43.5 |
|  | Very concerned | 43 | 25.1 | 29.3 | 72.8 |
|  | Extremely concerned | 40 | 23.4 | 27.2 | 100.0 |
|  | Total | 147 | 86.0 | 100.0 |  |
| Missing | System | 24 | 14.0 |  |  |
| Total | | 171 | 100.0 |  |  |

| **2.In the light to COVID-19, how often did you feel your sleep was restless?** | | | | | |
| --- | --- | --- | --- | --- | --- |
|  | | Frequency | Percent | Valid Percent | Cumulative Percent |
| Valid | Never | 11 | 6.4 | 7.5 | 7.5 |
|  | Occasionally | 29 | 17.0 | 19.7 | 27.2 |
|  | Sometimes | 51 | 29.8 | 34.7 | 61.9 |
|  | Often | 43 | 25.1 | 29.3 | 91.2 |
|  | Always | 13 | 7.6 | 8.8 | 100.0 |
|  | Total | 147 | 86.0 | 100.0 |  |
| Missing | System | 24 | 14.0 |  |  |
| Total | | 171 | 100.0 |  |  |

| **3.In the light to COVID-19, how often did you feel anxious that you might get infected with COVID-19 virus?** | | | | | |
| --- | --- | --- | --- | --- | --- |
|  | | Frequency | Percent | Valid Percent | Cumulative Percent |
| Valid | Never | 10 | 5.8 | 6.8 | 6.8 |
|  | Occasionally | 36 | 21.1 | 24.5 | 31.3 |
|  | Sometimes | 62 | 36.3 | 42.2 | 73.5 |
|  | Often | 25 | 14.6 | 17.0 | 90.5 |
|  | Always | 14 | 8.2 | 9.5 | 100.0 |
|  | Total | 147 | 86.0 | 100.0 |  |
| Missing | System | 24 | 14.0 |  |  |
| Total | | 171 | 100.0 |  |  |

| **4. In the light to COVID-19, are you concerned about contracting COVID -19 from providing patient care in the clinics?** | | | | | |
| --- | --- | --- | --- | --- | --- |
|  | | Frequency | Percent | Valid Percent | Cumulative Percent |
| Valid | Not Concerned | 2 | 1.2 | 1.4 | 1.4 |
|  | A little concerned | 36 | 21.1 | 24.5 | 25.9 |
|  | Concerned | 51 | 29.8 | 34.7 | 60.5 |
|  | Very concerned | 30 | 17.5 | 20.4 | 81.0 |
|  | Extremely concerned | 28 | 16.4 | 19.0 | 100.0 |
|  | Total | 147 | 86.0 | 100.0 |  |
| Missing | System | 24 | 14.0 |  |  |
| Total | | 171 | 100.0 |  |  |

| **5. In the light to COVID-19, are you concerned about contracting COVID -19 from attending classes in the faculty?** | | | | | |
| --- | --- | --- | --- | --- | --- |
|  | | Frequency | Percent | Valid Percent | Cumulative Percent |
| Valid | Not Concerned | 11 | 6.4 | 7.5 | 7.5 |
|  | A little concerned | 47 | 27.5 | 32.0 | 39.5 |
|  | Concerned | 46 | 26.9 | 31.3 | 70.7 |
|  | Very concerned | 25 | 14.6 | 17.0 | 87.8 |
|  | Extremely concerned | 18 | 10.5 | 12.2 | 100.0 |
|  | Total | 147 | 86.0 | 100.0 |  |
| Missing | System | 24 | 14.0 |  |  |
| Total | | 171 | 100.0 |  |  |

| **6.In the light to COVID-19, are you concerned about contracting COVID -19 from interacting with people in the faculty building?** | | | | | |
| --- | --- | --- | --- | --- | --- |
|  | | Frequency | Percent | Valid Percent | Cumulative Percent |
| Valid | Not Concerned | 3 | 1.8 | 2.0 | 2.0 |
|  | A little concerned | 45 | 26.3 | 30.6 | 32.7 |
|  | Concerned | 52 | 30.4 | 35.4 | 68.0 |
|  | Very concerned | 28 | 16.4 | 19.0 | 87.1 |
|  | Extremely concerned | 19 | 11.1 | 12.9 | 100.0 |
|  | Total | 147 | 86.0 | 100.0 |  |
| Missing | System | 24 | 14.0 |  |  |
| Total | | 171 | 100.0 |  |  |

| **7.In the light to COVID-19, are you concerned about about-your emotional health?** | | | | | |
| --- | --- | --- | --- | --- | --- |
|  | | Frequency | Percent | Valid Percent | Cumulative Percent |
| Valid | Not Concerned | 6 | 3.5 | 4.1 | 4.1 |
|  | A little concerned | 28 | 16.4 | 19.0 | 23.1 |
|  | Concerned | 30 | 17.5 | 20.4 | 43.5 |
|  | Very concerned | 45 | 26.3 | 30.6 | 74.1 |
|  | Extremely concerned | 38 | 22.2 | 25.9 | 100.0 |
|  | Total | 147 | 86.0 | 100.0 |  |
| Missing | System | 24 | 14.0 |  |  |
| Total | | 171 | 100.0 |  |  |

| **8. In the light to COVID-19, how often did you feel you were unable to control important things in your life?** | | | | | |
| --- | --- | --- | --- | --- | --- |
|  | | Frequency | Percent | Valid Percent | Cumulative Percent |
| Valid | Never | 3 | 1.8 | 2.0 | 2.0 |
|  | Occasionally | 26 | 15.2 | 17.7 | 19.7 |
|  | Sometimes | 49 | 28.7 | 33.3 | 53.1 |
|  | Often | 38 | 22.2 | 25.9 | 78.9 |
|  | Always | 31 | 18.1 | 21.1 | 100.0 |
|  | Total | 147 | 86.0 | 100.0 |  |
| Missing | System | 24 | 14.0 |  |  |
| Total | | 171 | 100.0 |  |  |

| **9. In the light to COVID-19, how often did you feel you could not cope with all of the things that you had to do?** | | | | | |
| --- | --- | --- | --- | --- | --- |
|  | | Frequency | Percent | Valid Percent | Cumulative Percent |
| Valid | Never | 5 | 2.9 | 3.4 | 3.4 |
|  | Occasionally | 21 | 12.3 | 14.3 | 17.7 |
|  | Sometimes | 57 | 33.3 | 38.8 | 56.5 |
|  | Often | 40 | 23.4 | 27.2 | 83.7 |
|  | Always | 24 | 14.0 | 16.3 | 100.0 |
|  | Total | 147 | 86.0 | 100.0 |  |
| Missing | System | 24 | 14.0 |  |  |
| Total | | 171 | 100.0 |  |  |

| **10. In the light to COVID-19, how often did you feel how often do you feel angry because things were outside of your control?** | | | | | |
| --- | --- | --- | --- | --- | --- |
|  | | Frequency | Percent | Valid Percent | Cumulative Percent |
| Valid | Never | 10 | 5.8 | 6.8 | 6.8 |
|  | Occasionally | 30 | 17.5 | 20.4 | 27.2 |
|  | Sometimes | 53 | 31.0 | 36.1 | 63.3 |
|  | Often | 34 | 19.9 | 23.1 | 86.4 |
|  | Always | 20 | 11.7 | 13.6 | 100.0 |
|  | Total | 147 | 86.0 | 100.0 |  |
| Missing | System | 24 | 14.0 |  |  |
| Total | | 171 | 100.0 |  |  |

| **11.In the light to COVID-19, how often did you feel stressed?** | | | | | |
| --- | --- | --- | --- | --- | --- |
|  | | Frequency | Percent | Valid Percent | Cumulative Percent |
| Valid | Never | 1 | .6 | .7 | .7 |
|  | Occasionally | 15 | 8.8 | 10.2 | 10.9 |
|  | Sometimes | 46 | 26.9 | 31.3 | 42.2 |
|  | Often | 57 | 33.3 | 38.8 | 81.0 |
|  | Always | 28 | 16.4 | 19.0 | 100.0 |
|  | Total | 147 | 86.0 | 100.0 |  |
| Missing | System | 24 | 14.0 |  |  |
| Total | | 171 | 100.0 |  |  |

| **12. In the light to COVID-19, how often did you feel anxious regarding the uncertainly about how long the current crisis will last?** | | | | | |
| --- | --- | --- | --- | --- | --- |
|  | | Frequency | Percent | Valid Percent | Cumulative Percent |
| Valid | Never | 1 | .6 | .7 | .7 |
|  | Occasionally | 20 | 11.7 | 13.6 | 14.3 |
|  | Sometimes | 41 | 24.0 | 27.9 | 42.2 |
|  | Often | 43 | 25.1 | 29.3 | 71.4 |
|  | Always | 42 | 24.6 | 28.6 | 100.0 |
|  | Total | 147 | 86.0 | 100.0 |  |
| Missing | System | 24 | 14.0 |  |  |
| Total | | 171 | 100.0 |  |  |

| **13. In the light to COVID-19, how often did you feel depressed?** | | | | | |
| --- | --- | --- | --- | --- | --- |
|  | | Frequency | Percent | Valid Percent | Cumulative Percent |
| Valid | Never | 27 | 15.8 | 18.4 | 18.4 |
|  | Occasionally | 38 | 22.2 | 25.9 | 44.2 |
|  | Sometimes | 43 | 25.1 | 29.3 | 73.5 |
|  | Often | 33 | 19.3 | 22.4 | 95.9 |
|  | Always | 6 | 3.5 | 4.1 | 100.0 |
|  | Total | 147 | 86.0 | 100.0 |  |
| Missing | System | 24 | 14.0 |  |  |
| Total | | 171 | 100.0 |  |  |

| **14.In the light to COVID-19, are you concerned about your housing situation after faculty re-opens?** | | | | | |
| --- | --- | --- | --- | --- | --- |
|  | | Frequency | Percent | Valid Percent | Cumulative Percent |
| Valid | Not Concerned | 20 | 11.7 | 13.6 | 13.6 |
|  | A little concerned | 31 | 18.1 | 21.1 | 34.7 |
|  | Concerned | 48 | 28.1 | 32.7 | 67.3 |
|  | Very concerned | 31 | 18.1 | 21.1 | 88.4 |
|  | Extremely concerned | 17 | 9.9 | 11.6 | 100.0 |
|  | Total | 147 | 86.0 | 100.0 |  |
| Missing | System | 24 | 14.0 |  |  |
| Total | | 171 | 100.0 |  |  |

| **15. In the light to COVID-19, are you concerned about the well being of your family?** | | | | | |
| --- | --- | --- | --- | --- | --- |
|  | | Frequency | Percent | Valid Percent | Cumulative Percent |
| Valid | A little concerned | 9 | 5.3 | 6.1 | 6.1 |
|  | Concerned | 30 | 17.5 | 20.4 | 26.5 |
|  | Very concerned | 43 | 25.1 | 29.3 | 55.8 |
|  | Extremely concerned | 65 | 38.0 | 44.2 | 100.0 |
|  | Total | 147 | 86.0 | 100.0 |  |
| Missing | System | 24 | 14.0 |  |  |
| Total | | 171 | 100.0 |  |  |

| **16. In the light to COVID-19, are you concerned about your social connection?** | | | | | |
| --- | --- | --- | --- | --- | --- |
|  | | Frequency | Percent | Valid Percent | Cumulative Percent |
| Valid | Not Concerned | 9 | 5.3 | 6.1 | 6.1 |
|  | A little concerned | 28 | 16.4 | 19.0 | 25.2 |
|  | Concerned | 65 | 38.0 | 44.2 | 69.4 |
|  | Very concerned | 29 | 17.0 | 19.7 | 89.1 |
|  | Extremely concerned | 16 | 9.4 | 10.9 | 100.0 |
|  | Total | 147 | 86.0 | 100.0 |  |
| Missing | System | 24 | 14.0 |  |  |
| Total | | 171 | 100.0 |  |  |

| **17. In the light to COVID-19, how often did you feel lonely?** | | | | | |
| --- | --- | --- | --- | --- | --- |
|  | | Frequency | Percent | Valid Percent | Cumulative Percent |
| Valid | Never | 22 | 12.9 | 15.0 | 15.0 |
|  | Occasionally | 43 | 25.1 | 29.3 | 44.2 |
|  | Sometimes | 46 | 26.9 | 31.3 | 75.5 |
|  | Often | 20 | 11.7 | 13.6 | 89.1 |
|  | Always | 16 | 9.4 | 10.9 | 100.0 |
|  | Total | 147 | 86.0 | 100.0 |  |
| Missing | System | 24 | 14.0 |  |  |
| Total | | 171 | 100.0 |  |  |


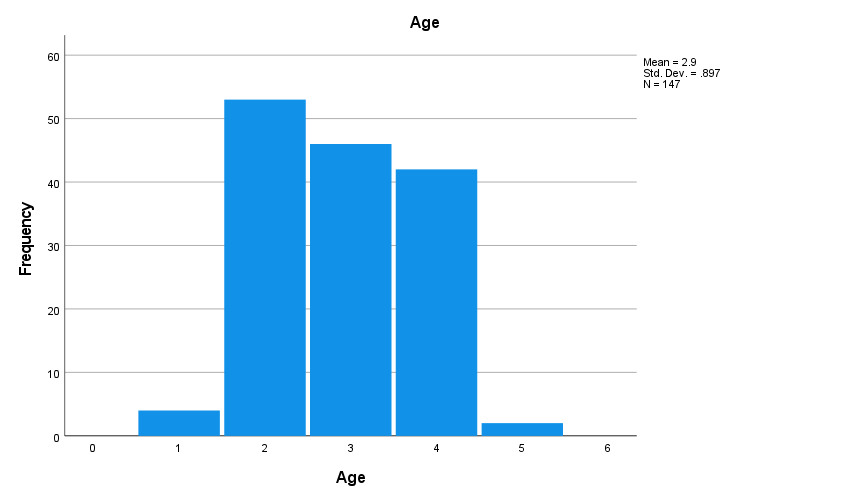


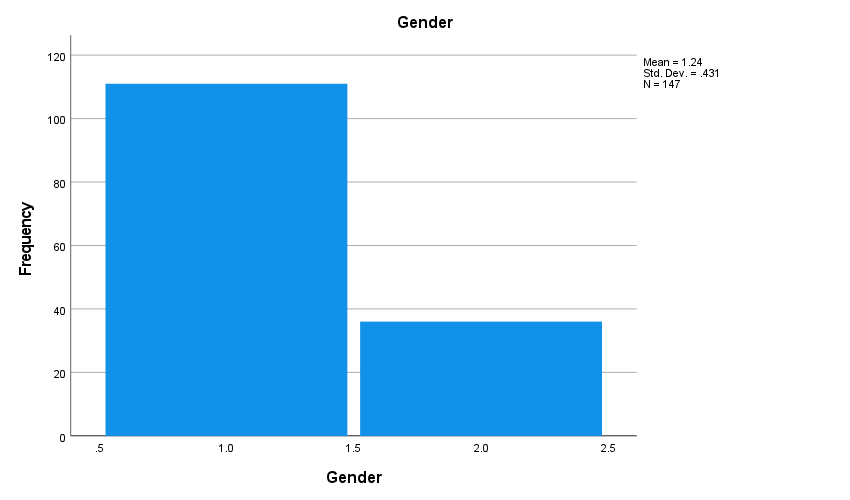


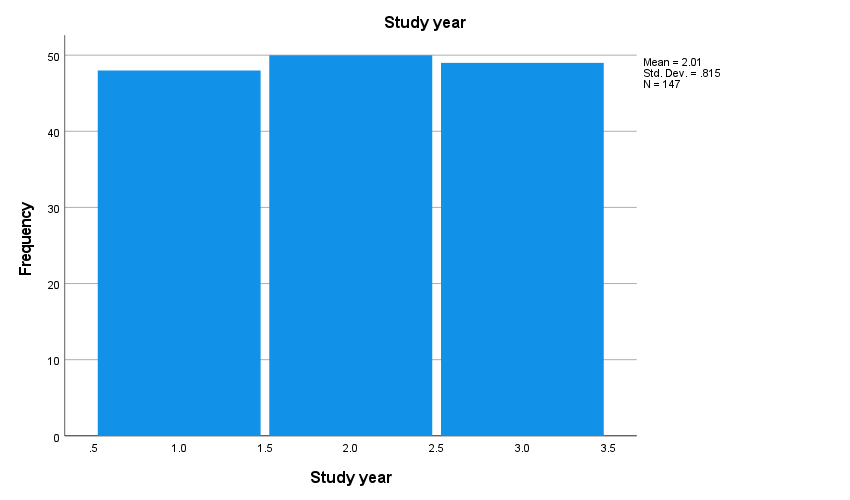


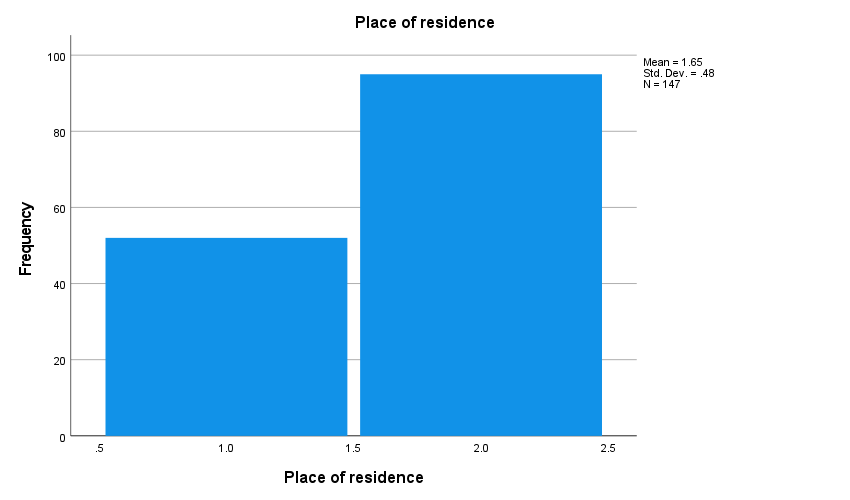


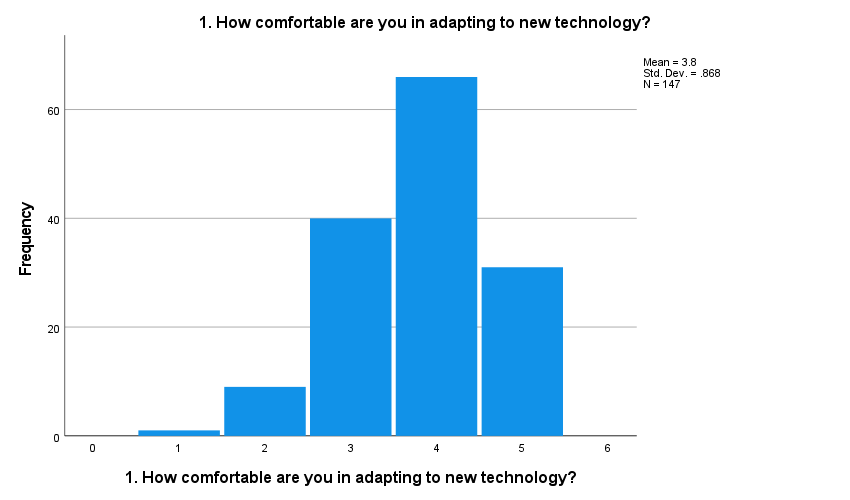


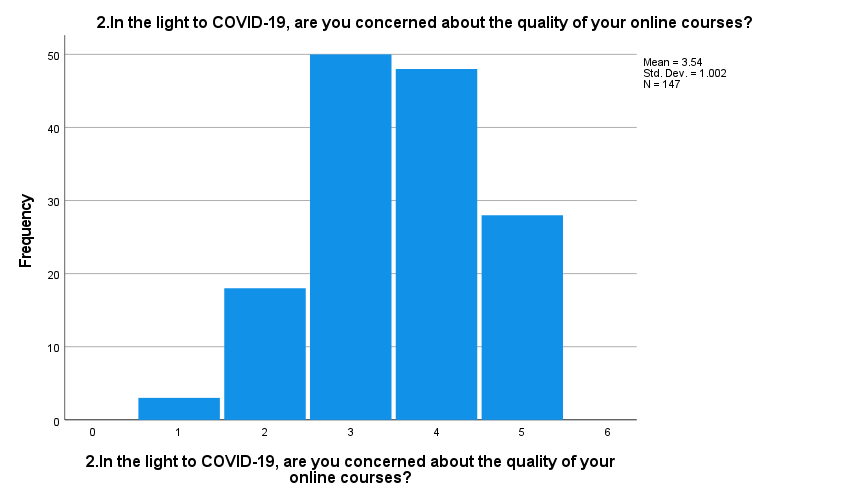


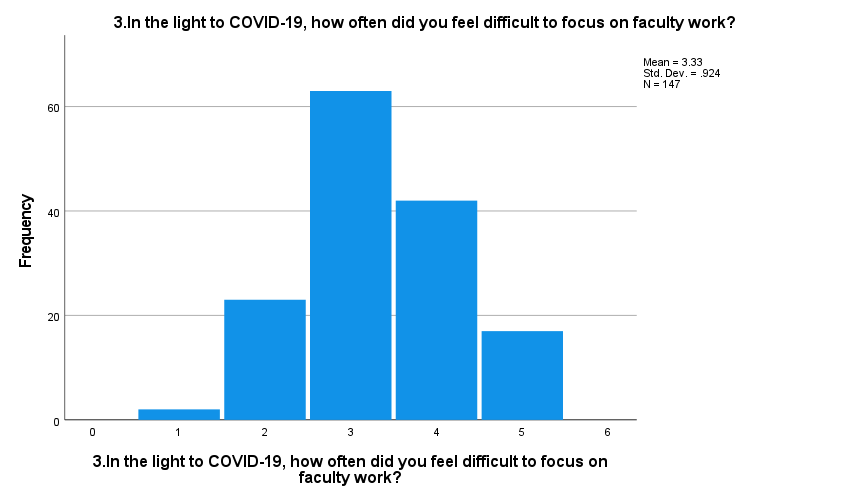


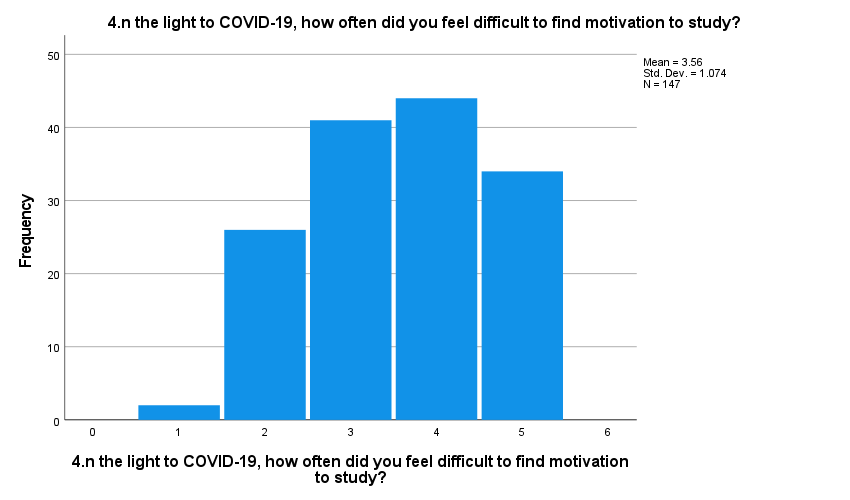


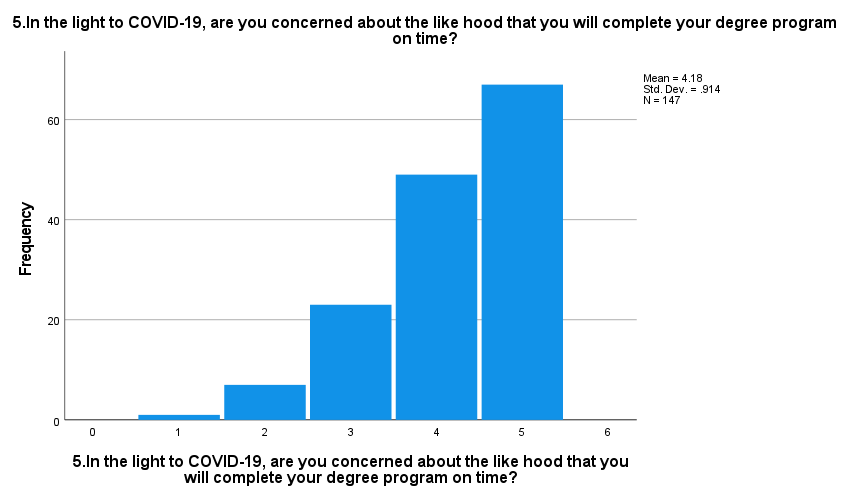


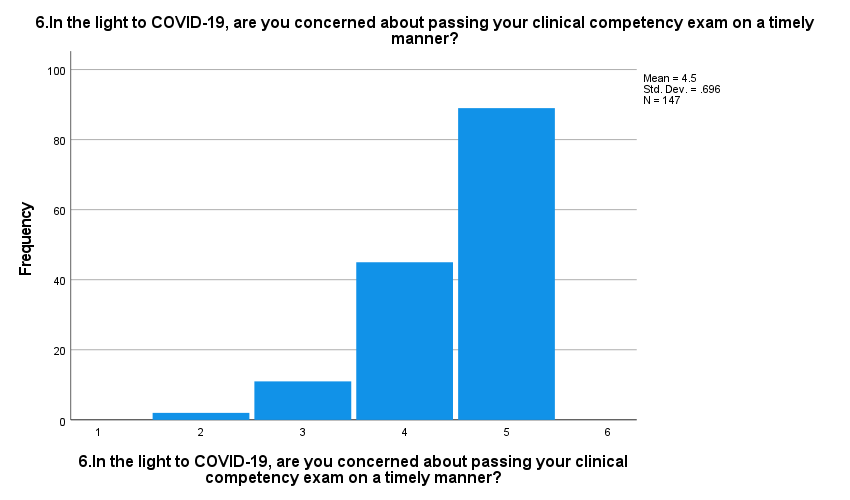


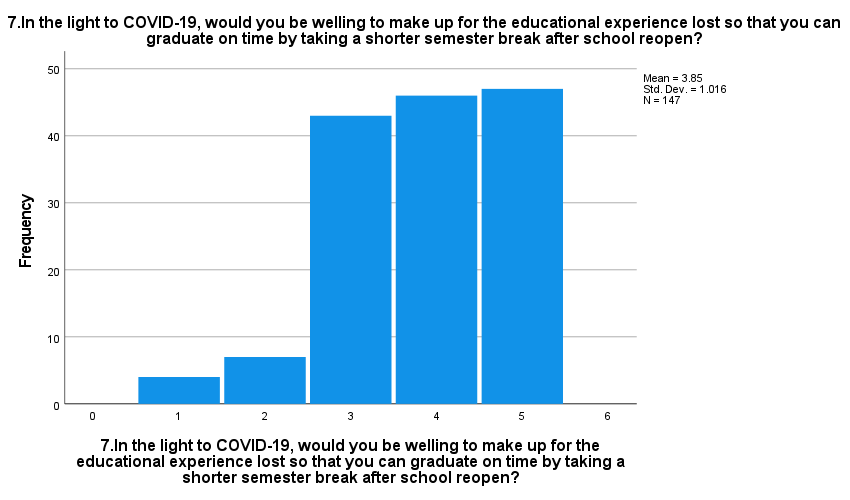


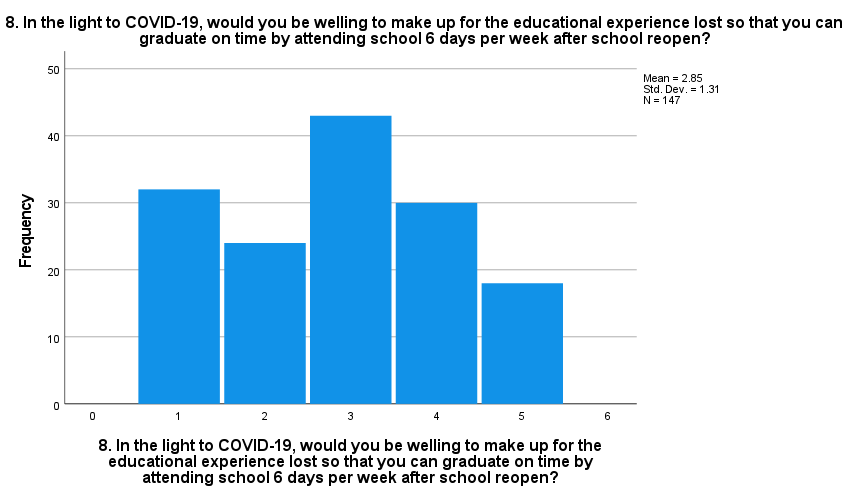


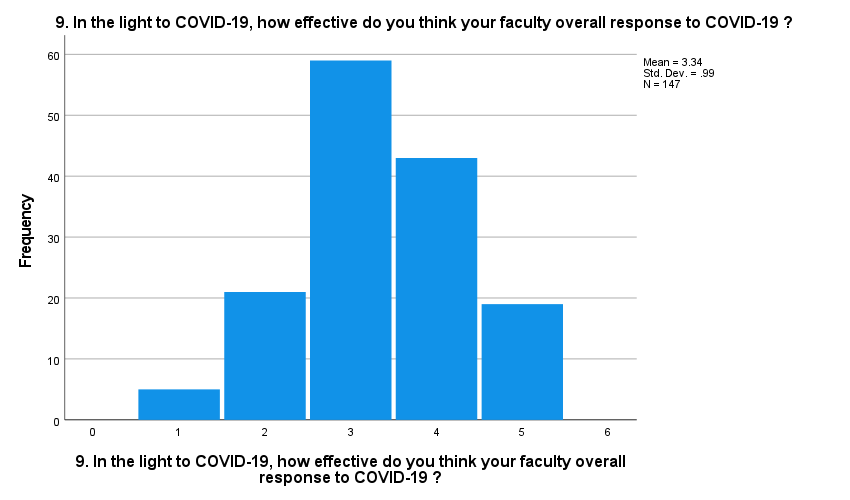


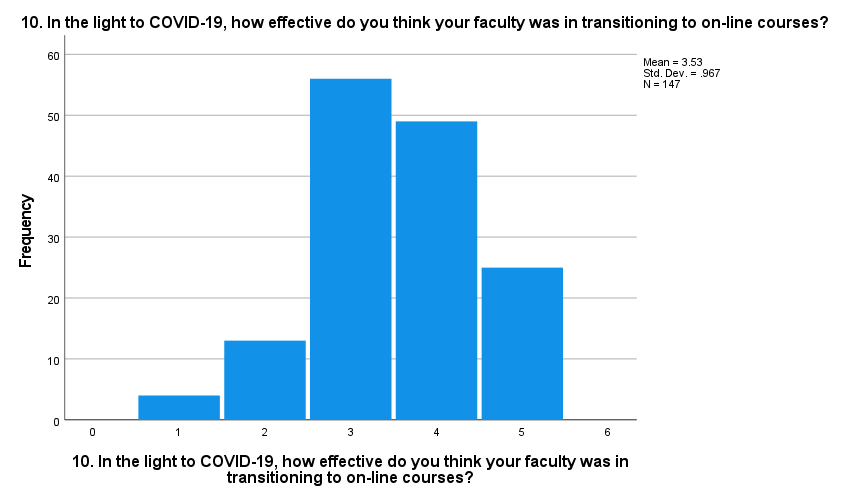


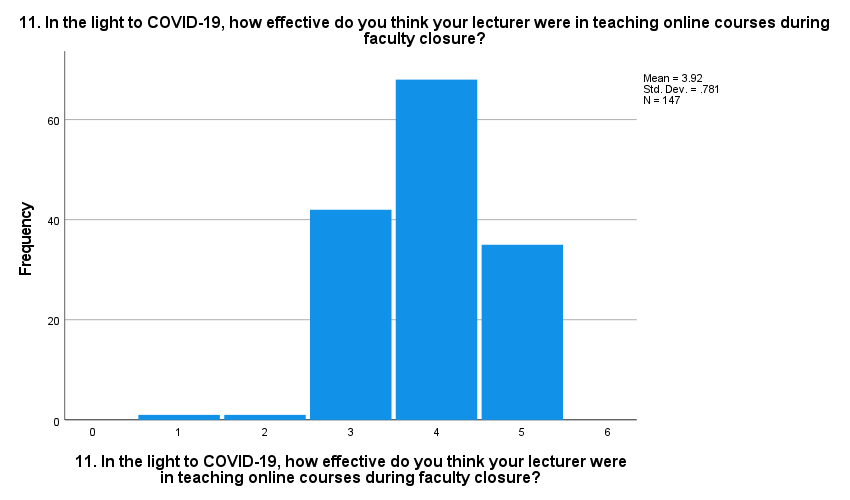


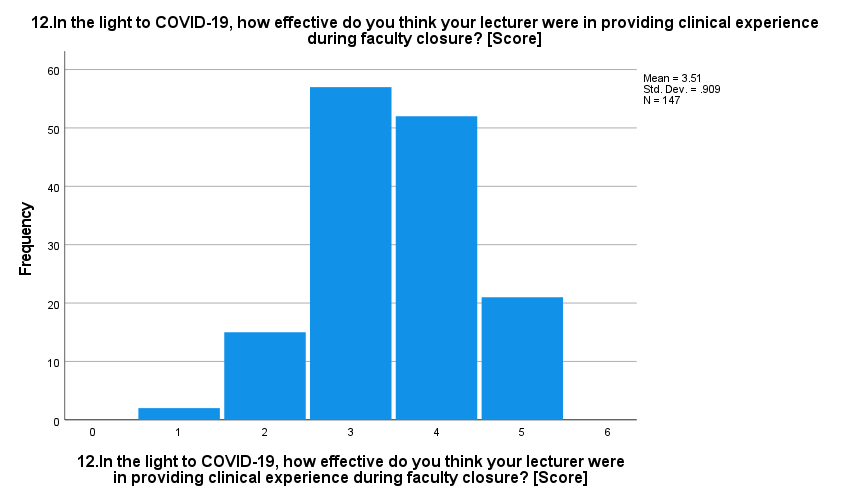


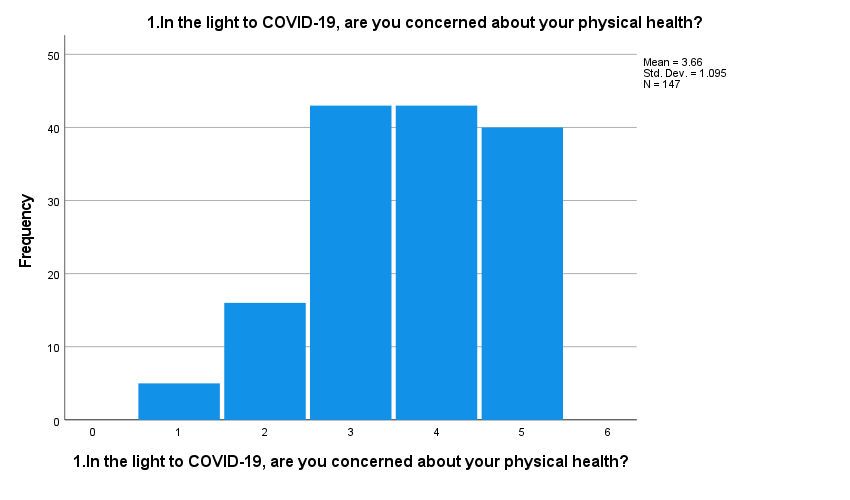


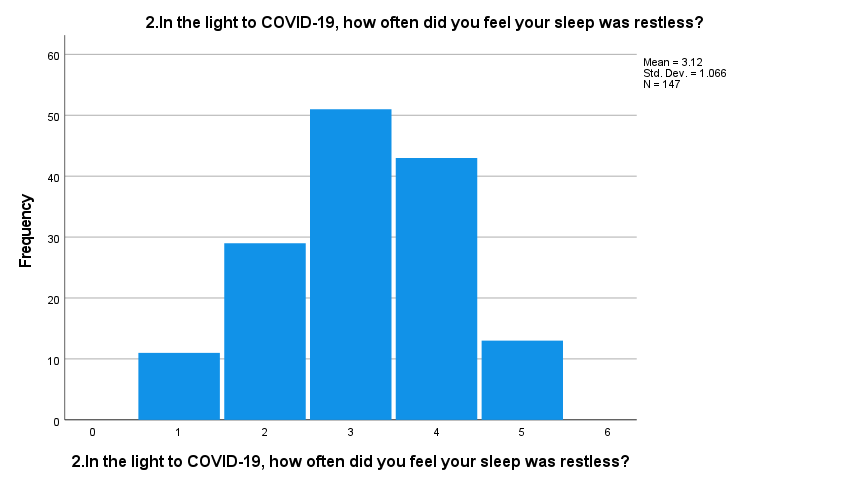


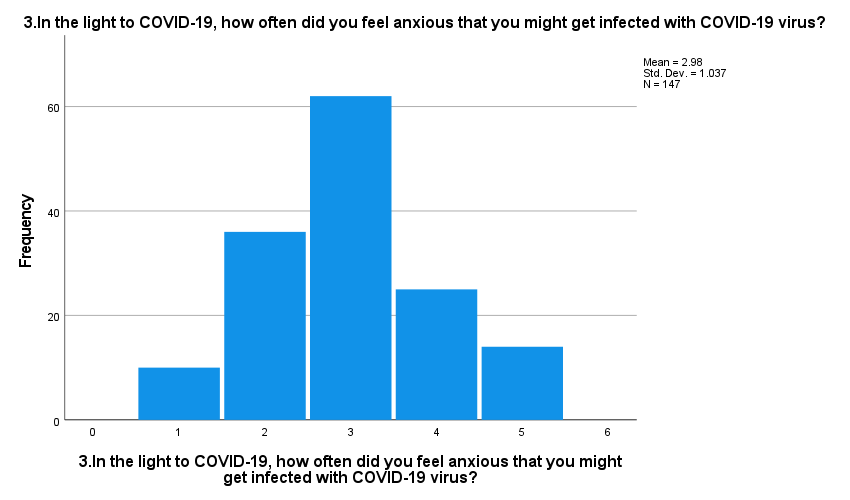


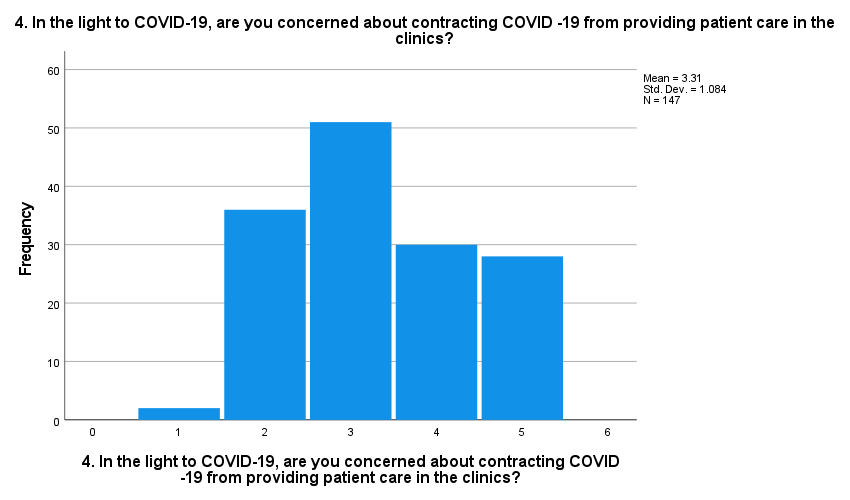


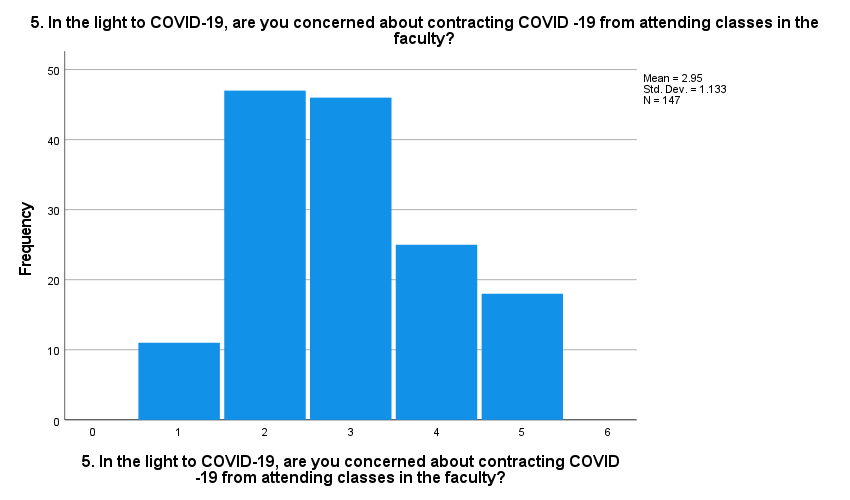


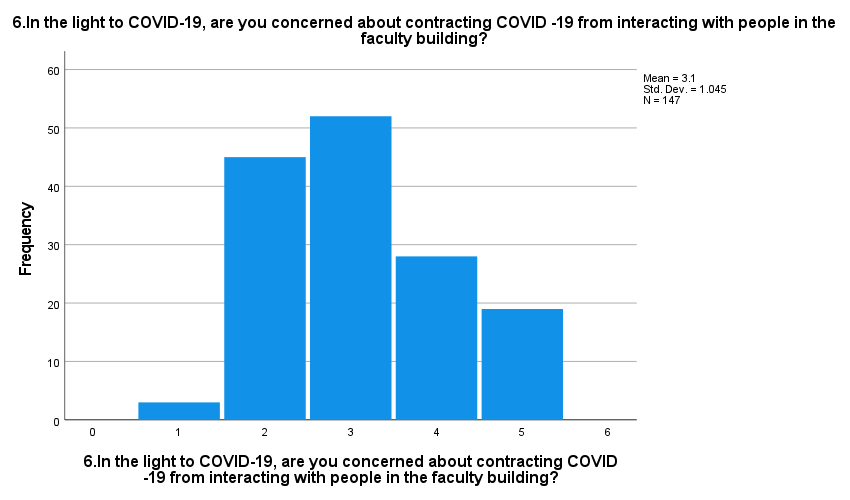


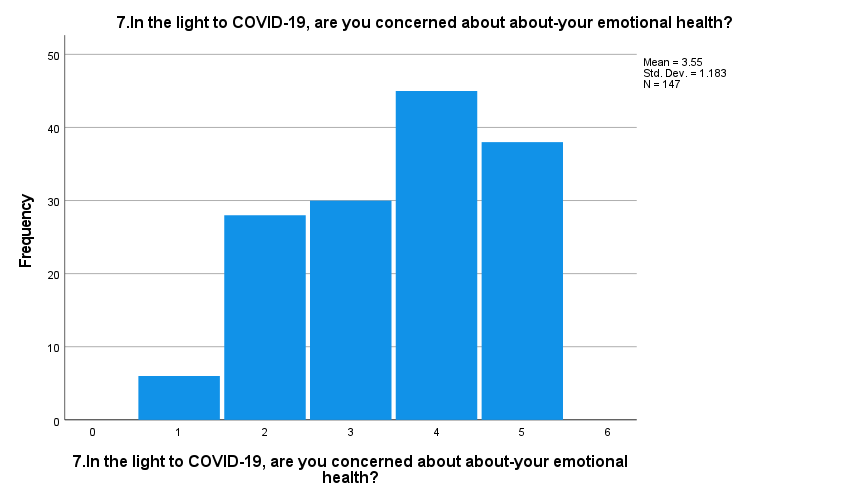


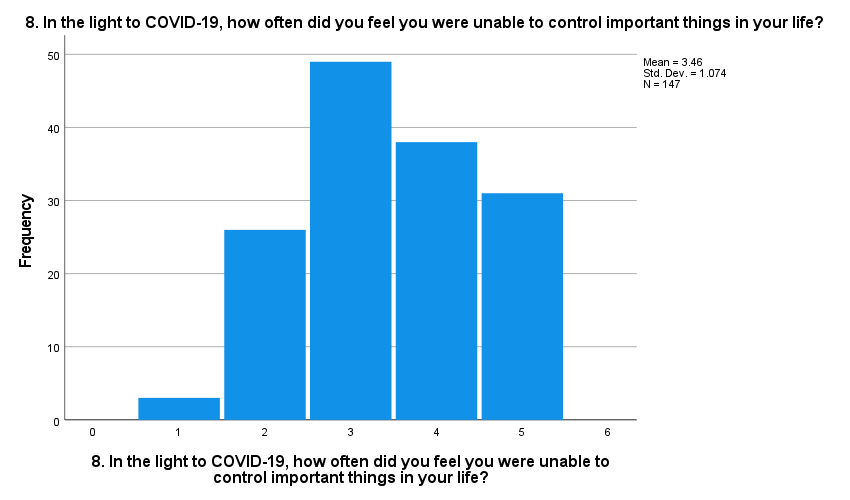


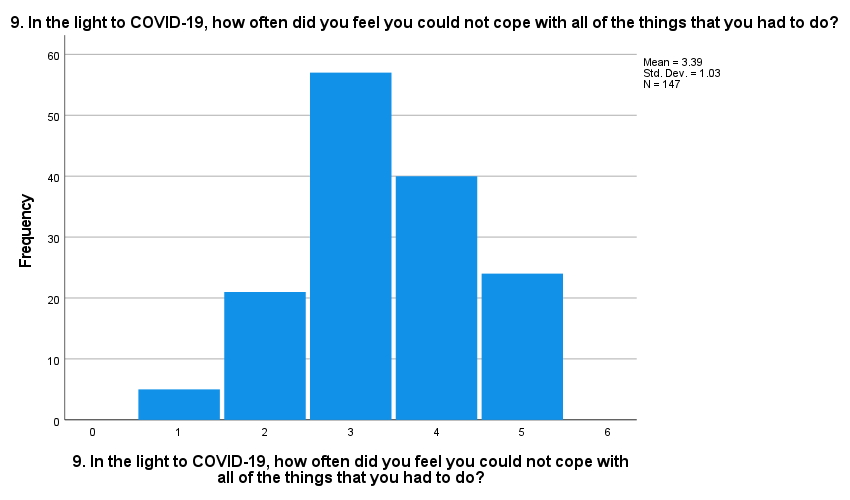


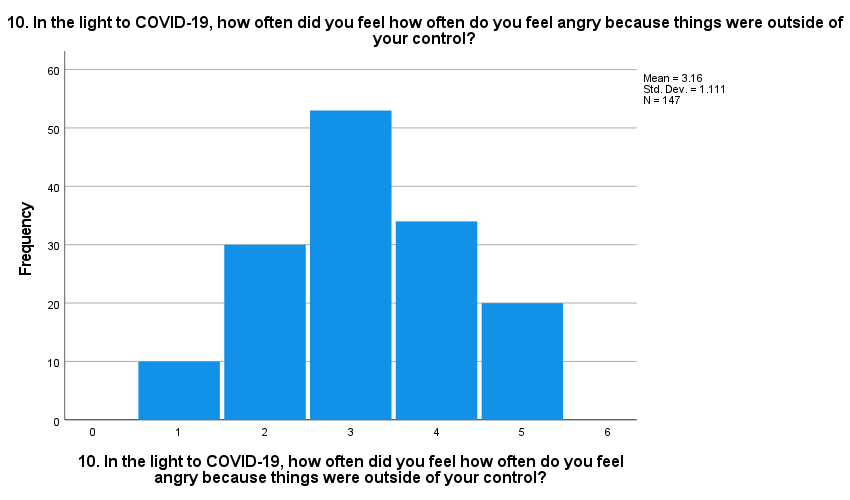


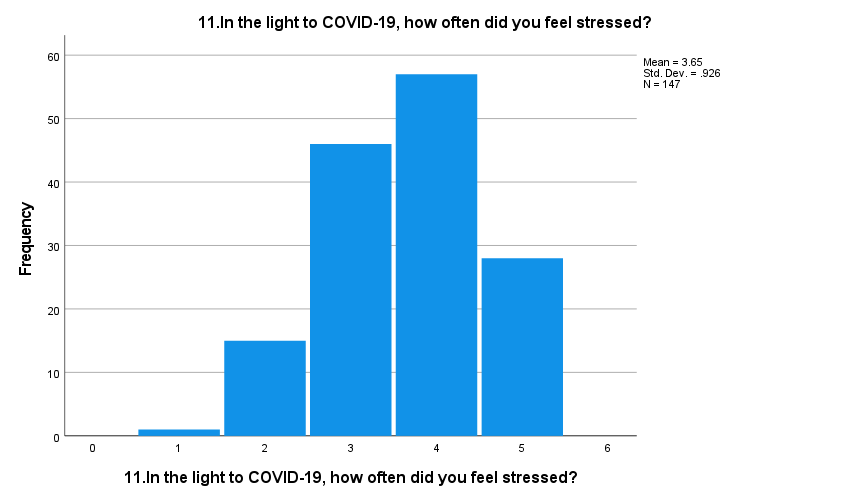


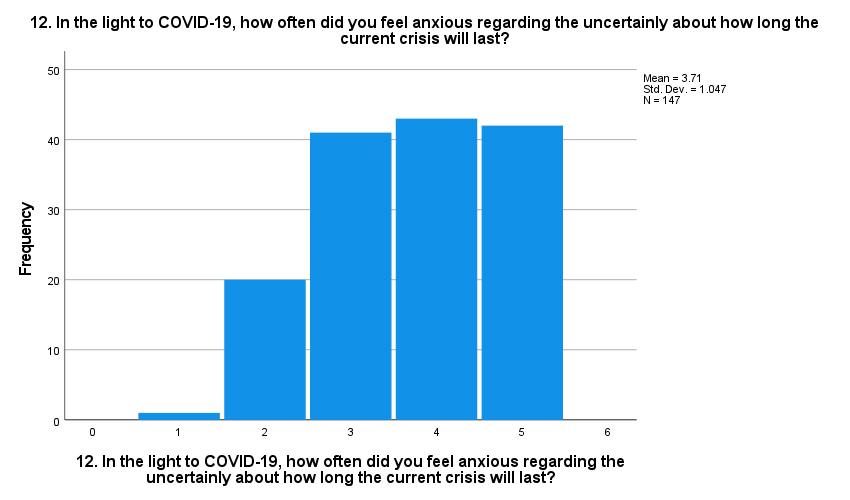


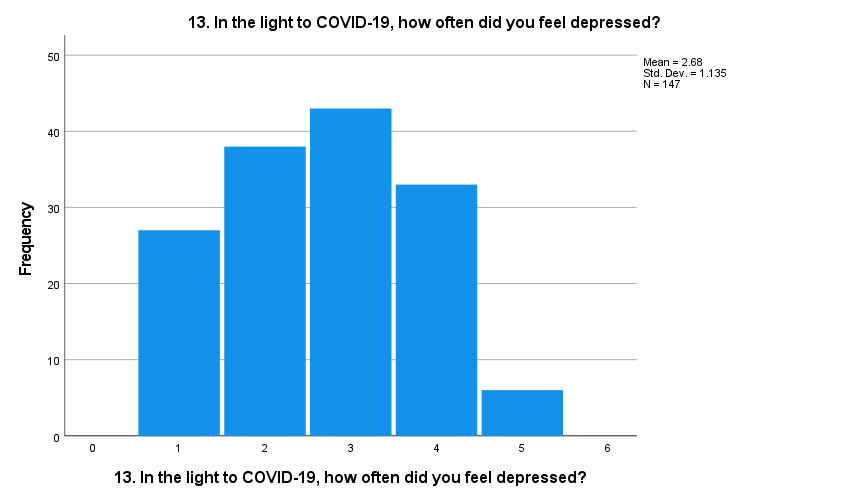


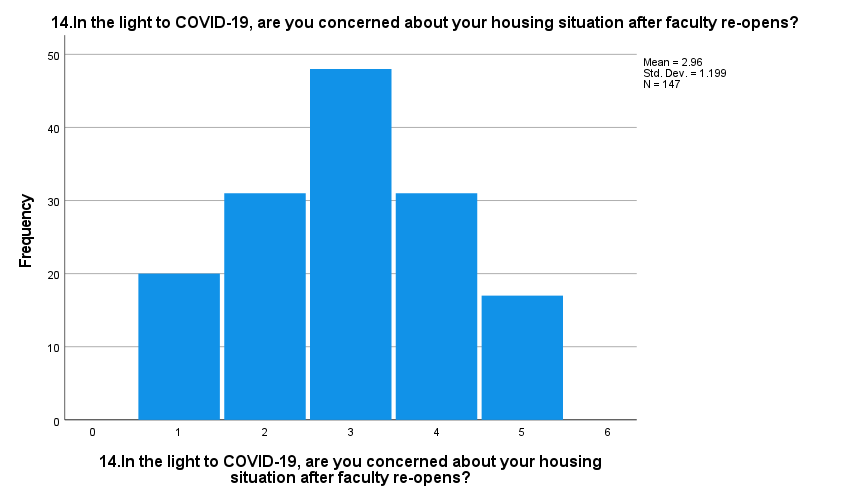


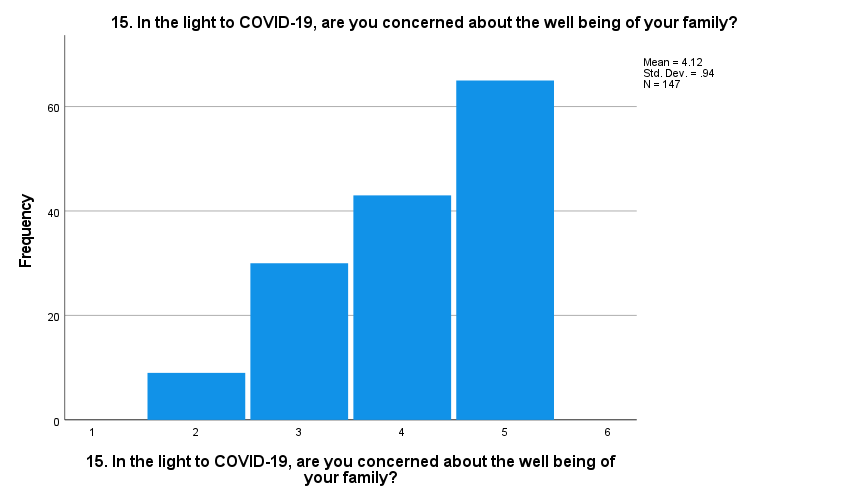


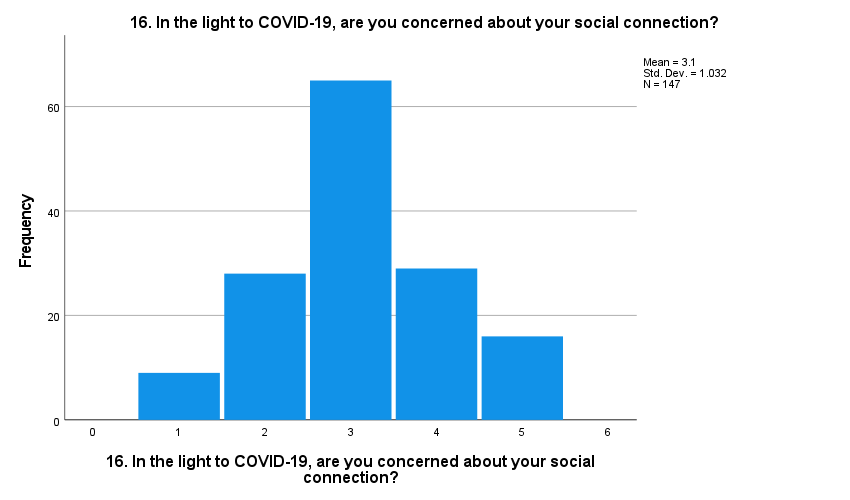


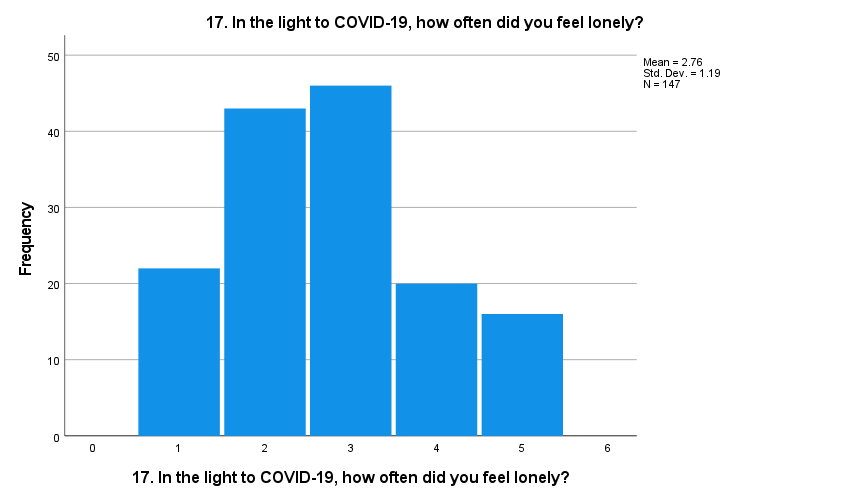

Supplement: S3 Appendix — (DOCX) [file pone.0270091.s003.docx]
